# Spontaneous Movements of a Computer Mouse Reveal Egoism and In-group Favoritism

**DOI:** 10.3389/fpsyg.2017.00013

**Published:** 2017-01-20

**Authors:** Norbert Maliszewski, Łukasz Wojciechowski, Hubert Suszek

**Affiliations:** ^1^Cardinal Stefan Wyszyński UniversityWarsaw, Poland; ^2^The Maria Grzegorzewska UniversityWarsaw, Poland; ^3^University of WarsawWarsaw, Poland

**Keywords:** “mouse” technique, Implicit Association Test, implicit attitude measure, altruism, altruistic behavior

## Abstract

The purpose of the project was to assess whether the first spontaneous movements of a computer mouse, when making an assessment on a scale presented on the screen, may express a respondent’s implicit attitudes. In Study 1, the altruistic behaviors of 66 students were assessed. The students were led to believe that the task they were performing was also being performed by another person and they were asked to distribute earnings between themselves and the partner. The participants performed the tasks under conditions with and without distractors. With the distractors, in the first few seconds spontaneous mouse movements on the scale expressed a selfish distribution of money, while later the movements gravitated toward more altruism. In Study 2, 77 Polish students evaluated a painting by a Polish/Jewish painter on a scale. They evaluated it under conditions of full or distracted cognitive abilities. Spontaneous movements of the mouse on the scale were analyzed. In addition, implicit attitudes toward both Poles and Jews were measured with the Implicit Association Test (IAT). A significant association between implicit attitudes (IAT) and spontaneous evaluation of images using a computer mouse was observed in the group with the distractor. The participants with strong implicit in-group favoritism of Poles revealed stronger preference for the Polish painter’s work in the first few seconds of mouse movement. Taken together, these results suggest that spontaneous mouse movements may reveal egoism (in-group favoritism), i.e., processes that were not observed in the participants’ final decisions (clicking on the scale).

## Introduction

Measurement of processes that are less controlled, automatized, or unconscious has been of particular interest in psychology from the very beginning. In experimental psychology, the division of processes into automatic vs. controlled appeared in the 1970s in research on attention (Shiffrin and Schneider, 1977). It was proposed that automatic processing requires no effort, is done unconsciously (though it may also be conscious), beyond one’s attention and intentional control. Basically, it does not depend on the cognitive resources and motivation to make an effort. Controlled processes, on the other hand, are executed when the subject draws attention to a certain object and makes a cognitive effort. They are sequential, conscious, and dependent on the person’s goals and intentions. They, therefore, require motivation and cognitive resources.

In the late 1970s the controlled/automated process distinction that originated from cognitive psychology was adopted into the field of social psychology, especially in the domains of attitudes and altruism research.

Research conducted to understand how attitudes operate contributed to the development of two competing paradigms. Within the first of them, researchers assumed that attitudes are constructed in a specific situational context, e.g., they are inferred from current thoughts and feelings (Tesser, 1978), mood (Schwarz and Clore, 1983), and behavioral observations (Bem, 1972). The basic assumption behind the second paradigm is that attitudes constitute relatively permanent evaluations stored in the memory (Fazio, 1986). In this paradigm, Fazio et al. (1986) observed that the mere presence of an object may automatically activate its evaluations as stored in the memory. This process occurs effortlessly and without the participation of consciousness (Fazio et al., 1989; Bargh et al., 1992; Blascovich et al., 1993). This phenomenon has allowed for a better understanding of the influence of attitudes on behavior. Fazio (1986) and Fazio and Towles-Schwen (1999) called it a spontaneous process because it was activated by mere contact with the object of the attitudes. Attitudes serve as orienting values (Roskos-Ewoldsen and Fazio, 1992), i.e., as direct attention of the subject on the objects, which should either be avoided or approached.

In the 1980s an attempt to reconcile the results of these two lines of research, i.e., attitude as a temporary structure vs. a record in the memory, resulted in a dual understanding of attitudes. It was assumed that an attitude is differently expressed in the two systems, e.g., reflective vs. automatic, systematic vs. heuristic, and conscious vs. unconscious (Chaiken and Eagly, 1983; Petty and Cacioppo, 1984; Bargh, 1999; Chaiken and Trope, 1999; Smith and DeCoster, 2000). The division between two kinds of attitudes, each connected with a distinct system, was introduced by Greenwald and Banaji (1995), who proposed the notion of implicit attitudes. They defined it as “a trace of past experience that influences favorable or unfavorable feelings, thoughts and actions toward social objects, although this trace of memory remains introspectively unidentified or identified inaccurately” (p. 8). Wilson et al. (2000) assumed that implicit attitude is different from explicit attitude, so the subject can have two, often evaluatively different, attitudes toward the same object (e.g., I like cigarettes; cigarettes cause cancer). Implicit attitude is related to the automatic system, while explicit attitude is related to the reflective system (cf. Gawronski and Bodenhausen, 2006). A large body of research suggests that if the implicit attitude is formed and operates in the “automatic” system, it is strongly associated with spontaneous behaviors that are undertaken without the effort and control of the subject (Dovidio et al., 1997; Marsh et al., 2001; McConnell and Leibold, 2001; Perugini, 2005). The hypothesis regarding the impact of implicit attitude in automatic conditions is also supported by neuroimaging (fMRI) studies (Cunningham et al., 2004). These suggest that the first few seconds of any reaction can be regulated by the implicit attitude, i.e., it is automatically activated in the presence of the object of an attitude and it will determine the first reactions to the object (i.e., at least in a short period of time; the first second). The subject needs time to apply explicit attitudes (cf. Wilson et al., 2000). Explicit attitude can take over the regulatory role only after some time. In this article we verified the hypothesis regarding the dynamics of the impact on the behavior of implicit attitudes. A similar research problem was identified in the debate over whether intuition promotes either altruism or egoism.

### Dual-Process Framework of Altruistic vs. Egoistic Behavior

Apart from the usefulness of the dual-process framework in the area of attitude research, its application proved to be fruitful in the study of mechanisms underlying altruistic vs. egoistic behaviors.

The studies were aimed at answering the basic question whether people are intuitively inclined to cooperate or whether they behave selfishly on the automatic level and altruism requires time and cognitive resources (Rand et al., 2012; Capraro and Cococcioni, 2015; Corgnet et al., 2015).

One approach suggests that pursuing self-interest is an automatically activated goal, while pro-social behavior requires cognitive resources. Traditional economic and evolutionary models also assume that people are predisposed toward selfishness (Dawkins, 1976; Myers, 1983; Axelrod and Hamilton, 1984). However, several researchers have reported results that directly oppose this commonly held belief.

Rand et al. (2012) showed that intuition promoted by cognitive load increased generosity in resource allocation. Furthermore, forcing subjects to decide quickly increased contributions, whereas instructing them to reflect and forcing them to decide slowly decreased contributions. Finally, an instruction that primes subjects to trust their intuitions increased contributions as compared to an instruction that promotes greater reflection. To explain these results, Rand et al. (2012) proposed that cooperation is intuitive because cooperative heuristics are useful in daily life in which cooperation is advantageous. If decisions were based on a more elaborated thinking process, pursuing self-interest would be dominating.

The conclusion that people are intuitively cooperative in social dilemma games was challenged by several researchers. Tinghög et al. (2013) studied the robustness of the findings of Rand et al. (2012) in a series of experiments involving about 2,500 participants. None of the experiments confirmed Rand et al.’s (2012) findings, indicating that their result was an artifact of excluding about 50% of subjects who had failed to respond on time. Results of the studies by Verkoeijen and Bouwmeester (2014) also failed to reveal an intuitive-cooperation effect.

One of the ways to explain the differences between these opposite results (i.e., showing or not that intuition promotes altruistic behavior) is to assume that they captured different automatic reactions; for instance, both Pavlovian (inborn) and habitual (learned) processes may operate automatically and sometimes may give different results (Gęsiarz and Crockett, 2015). The habitual system emits actions based on the reinforcement history, and the Pavlovian system promotes reactions based on evolutionarily prescribed priors.

However, Pavlovian or habitual processes do not always promote a particular kind of behavior (pro-social or pro-self). Some pro-social processes are triggered by old, evolutionary mechanisms embedded in the Pavlovian system; nonetheless, pro-social behaviors can also be learned and automated, e.g., cooperative heuristics.

An additional factor that should be taken into consideration when explaining the differences between the results of the relationship between automated behavior and pro-social behavior is time dynamics. In a study by Piovesan and Wengström (2009), decisions made quickly were pro-self; those made after some time were more pro-social (the money was divided more equally). We interpreted the results as that the quick decisions require very simple mechanisms, e.g., the decisions may be the result of an automatically activated goal, e.g., pursuing self-interest. Much of the goal pursuit is now known to occur without one’s awareness or intent, i.e., it can be automatically triggered (Chartrand and Bargh, 1996; Gollwitzer, 1999). Moreover, currently activated goals may impact other automated reactions, i.e., either inhibit or enhance them (Gollwitzer, 1999). Not only goals but also habitual processes and norms are automatically activated. Acting according to the norm of fairness by providing an equal endowment is an example of this mechanism (Konow, 2010). Following this interpretation, with time people may change their automatic responses from pro-self to pro-social ones. Piovesan and Wengström (2009) observed that the longer it took to make a decision, the more pro-social it was. This interpretation became the rationale to set a new research goal, i.e., to check whether there exists a dynamics of decision making on the automatic level of processing (first a pro-self decision is made, and with time it shifts toward a pro-social one).

We assumed that different parallel mechanisms of the decision-making process during social interactions may operate on an automatic level, but it is not necessarily possible to capture the influence in the frame of dual-process theory because in previous research only the final, automatic decisions were measured. In the following project we examined the temporal dynamics of this process by checking both the first spontaneous reactions and the final decisions. It was predicted that the first spontaneous behavior would be more egoistic (implementation of the self-interest goal) and then became more altruistic (which was studied and reported in dual-process perspective research, comp. Rand et al., 2012, 2014).

### Indirect Measures and the Necessity of Time Dynamics

Explicit attitudes are measured directly, by using a questionnaire. The subjects are asked directly about the relationship to the object (direct measurement). Responses are expressed, among others, as on the Likert scale, Thurston’s scale or semantic differential. This measurement method assumes that the person wants and can express his/her attitude.

Direct measurements are not applicable in the case of implicit attitudes, i.e., one cannot directly investigate the relationship to the object while the participant is not aware of this relationship. The measurement of implicit attitudes requires the use of indirect measures.

Methods for indirect measurement of attitudes became popular in the 1970s and 1980s (Fazio et al., 1986; Bargh et al., 1992; Bargh and Chartrand, 2000). The most popular methods were derived from the implicit memory test (implicit memory), which was based on the phenomenon of priming (e.g., Neely, 1977; for a review, see Lewandowsky et al., 1989). An example of such measures of implicit attitudes is affective priming (e.g., Fazio et al., 1986, 1995; De Houwer et al., 1998; Fazio, 2001; Fazio and Olson, 2003).

Another way of estimating implicit attitudes is to study categorization processes, which are the basis of the Implicit Association Test (abbreviated as IAT; Greenwald et al., 1998). An indicator of implicit attitudes in the test is the difference between the time of classifying objects into categories when categories are arranged in pairs that are affectively consistent or inconsistent (IAT description, see Study 2); for example, the person performing the IAT is biased and has a negative association with Jews. If in the IAT a Jewish person is paired with unpleasant stimuli and a Polish person with pleasant stimuli, the categorization of objects is easy and the prejudiced person performs the tasks quickly. On the other hand, if the task is inconsistent (“a pleasant Jewish” and “an unpleasant Polish person”), then the categorization is difficult and the response time is longer. On the basis of the difference in the reaction to these two tasks, both the character (positive vs. negative) and strength of the attitude can be determined. The measurement is indirect, as the examined person does not know that the difference in the reaction time is an indicator of his/her attitude.

Another group of techniques of indirect measurement of implicit attitudes is based on recording the approach vs. avoidance reaction toward a given object. The Implicit Association Procedure (IAP; Schnabel et al., 2006) may serve as an example. Similar to the IAT, the IAP aims to assess automatic associations between concepts (e.g., “pleasant,” “Polish,” “Jewish”) through a series of discrimination tasks. Instead of categorizing stimuli by using a key on the keyboard, participants, in front of the monitor, express their attitudes to the object on the screen by using a joystick. If the object is close to them, they move the joystick toward themselves (approach), if they prefer to avoid the object, they move the joystick away from themselves. The IAP triggers the automatic approach and avoidance behavior via two joystick movements (pushing it away or toward a target; Neumann et al., 2004).

A disadvantage of the above methods is that they do not allow us to study the dynamics of the object of evaluation. The “mouse method” allows us to perform this type of measurement (Vallacher and Nowak, 1999). In the method, subjects are asked to express their feelings, attitudes, or evaluations by moving a computer mouse, e.g., around a point on the screen that symbolizes the evaluated object. The method is similar to methods that were used in the 1970s to assess the attractiveness of movies, i.e., the more the person liked a part of the movie, the more he/she squeezed a “pear,” and if the following scenes were “worse,” he/she weakened his/her squeeze on the pear. In the mouse method a person expresses an opinion which is being recorded. Then the person evaluates the statement by using mouse movements, i.e., the more negative the attitude, the further from the center of the screen one places the mouse pointer. As a consequence, not only is the final assessment recorded (the point that the person chose), but also the dynamics of the change of that assessment (the trace of the pointer). The mouse method was meant to describe complex dynamics of mental processes.

Mouselab, a computer-based information board, provides data regarding strategy classification in decision making (Johnson et al., unpublished), but generally it can also be used in human information processing research (Spivey et al., 2005; Glöckner and Betsch, 2008; Koop and Johnson, 2011). The method involves the presentation of options in a covered information matrix. Participants uncover the outcomes of choice options by moving the mouse cursor onto boxes. The information search proceeds in steps which can be recorded by the program and which serve as cues to identify decision strategies.

The present research introduces a modification of the two “mouse methods.”

## Overview of the Research

The purpose of the project was to assess whether the first spontaneous movements of a computer mouse when making an assessment on a scale presented on the screen may express the implicit attitudes of the respondents. The subjects perform a task on a computer screen, believing that only their final assessment is recorded, e.g., the value they end up selecting on the scale. They are not informed that the mouse movements they perform while arriving at the final decision are continuously being recorded. It is expected that the spontaneous mouse movements preceding the final decision may express the participants’ implicit reactions/attitudes.

In Study 1, the participants divided small sums of money between themselves and the experimenter’s coworker. They could split the money in a way that was more or less selfish. It was predicted in our research that spontaneous mouse movements on a scale would reveal more selfish distribution of the money in the first few seconds. First the spontaneous movements would be the result of an automatically activated goal, i.e., pursuing the self-interest. Then, after a few seconds, the mouse movements on the scale became more altruistic, because i.e., people act according to the norm of fairness.

In Study 2, implicit attitudes were measured with the IAT (attitudes toward Poles vs. Jews). We predicted a significant association between implicit attitudes (IAT) and a spontaneous evaluation of the objects (a painting made by a Pole vs. a Jew) using a computer mouse. We assumed that the first few seconds of any reaction are regulated by implicit attitudes. It is automatically activated in the presence of the object of an attitude and it will determine the first reactions to the object. The subject needs time to apply explicit attitudes and there is a lag before he/she can take over the regulatory role.

## Study 1

The definition of egoism and altruism that was applied in this project was based on the Social Orientations conception (McClintock, 1972). It is based on game theory. Our research is coherent with this tradition and its assumptions, so we defined egoism (individualism) as care for one’s own welfare without considering the welfare of others. A perfect egoist is a person whose weight attached to the welfare of the self is equal to 1, and to the welfare of others is equal to 0. Altruism was defined as care for the welfare of others without considering welfare of the self. A perfect altruist is a person whose weight attached to the welfare of the self is equal to 0 while to the welfare of others it is equal to 1. The concept is often measured by asking people to distribute goods between themselves and another person. Frequently, money is used as a good and the participant can divide it between him/herself and his/her partner. In Study 1, a Dictator Game was chosen. In it, two participants are paired. One of them receives an amount of money and is instructed to divide the money between him/herself and his/her partner. The size of the dictator’s “donation” is a measure of altruism, or the degree to which one is motivated to pursue the collective interest.

In the Dictator Game, intuition was promoted by cognitive load (Cornelissen et al., 2011). Cognitive loads are elicited by using distraction manipulation. Intuition is also promoted by time pressure. Rand et al. (2012) asked participants to reach their decision quickly (within 10 s). They did not have enough time to think about their decisions, therefore the decisions were expected to be made intuitively. We used both distraction manipulation and time pressure to induce intuitive decision making (group 1). Under conditions of cognitive load and restricted time for reflection, we wanted to obtain the most automatic and simplest first reactions in the Dictator Game. No time pressure and no cognitive load conditions promoted a deliberative decision process (group 2).

Processes which constitute behavior can be categorized on a scale from the most complex to the most automatic and simplest (Nowak and Vallacher, 1998). Each complex adaptive system, such as a human being, may pursue goals with varying degrees of complexity depending on the availability of resources. In the case of greater availability of resources, the optimal solution may incorporate more complex processes, i.e., processes taking into account the effects of the action, an assessment of the social environment, or group norms. When the cognitive resources of an individual are limited (due to the impact of distractors, a lack of time), the simplest and most automatic processes are selected, such as an automatically activated goal of pursuing the self-interest. First, we predicted that in the first few seconds a person would behave in a selfish manner. With the passage of time the level of altruistic behavior as measured by the mouse procedure should increase. Then the norm of fairness is applied, but its use may require time and resources. Thus we predicted that this relationship should be stronger when the distractor worked and the application of the norm of fairness would take more time.

### Method

#### Subjects

A total of 66 participants took part in the study. They were recruited at the campus of Warsaw University (Faculty of Psychology; age *M* = 19.48, *SD* = 0.662). The study took place in a computer room at the Faculty of Psychology. On average, each participant required 15 min to complete the study.

#### Study Design

The first independent variable (between-group) was described as the conditions for distributing the money (with or without a distractor). The second independent variable (within-group) was the time in which the reaction was measured (spontaneous mouse movements were measured every 250 ms). The dependent variable was the division of money (the position of the cursor on the scale of graphics at the time the person was pondering and the final decision).

#### Tools and Materials

**FIGURE 1:**
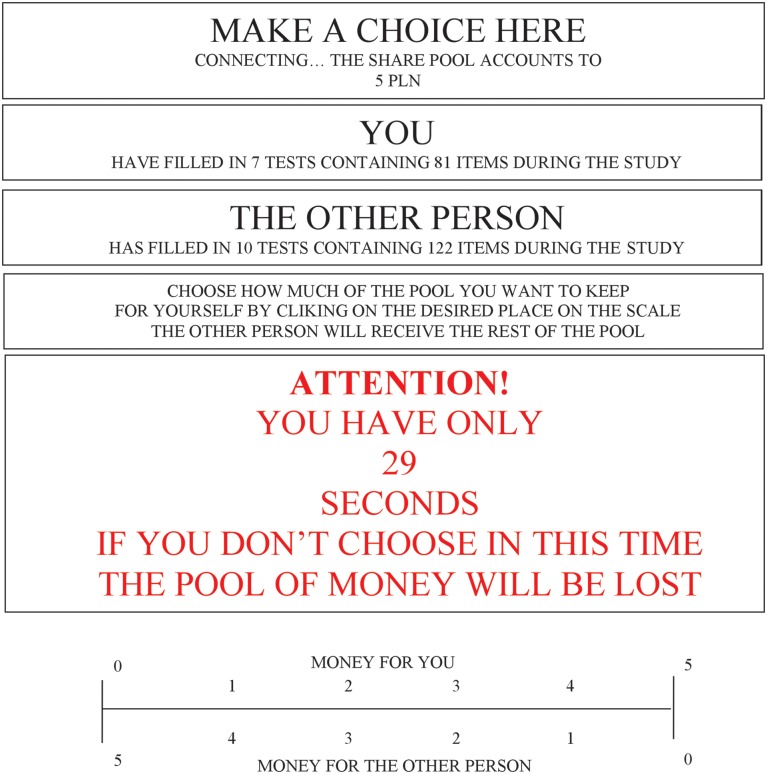
**Screen that served to distribute the money (the person pondered what sum to select and then clicked on it) under “the distractor.”**.

The only tool used in the study was the “mouse paradigm.” This is a computer method. At the bottom of the screen the subjects saw a graphic scale distribution of money (0–5 PLN) (see **Figure 1**). Clicking on this scale, the participants decided how much money from a pool of 5 PLN they could spend on themselves and how much on another (unknown) person. The higher the amount of money the respondents chose for themselves, the more their behavior was characterized as more selfish. We recorded not only the final decisions (clicks), but also the moves of the computer mouse while the participant was pondering the selection (the positions of the cursor on the scale were measured every 250 ms, which is a value expressed in pixels).

The distractor was a dynamic change in the background color combined with a strong (with a frequency of every 50 ms) flickering of the screen. In addition, information (in red color) about the possible loss of money was displayed if the decision was not made in 40 s. The number of seconds remaining was displayed on the screen.

A total of 40 s was the limit to provide an answer under the distraction condition. The subjects could respond in any time period before the time limit. There was no time limit under the no cognitive load conditions (subjects could deliberate for as long as they wanted). Participants could see the (x, y) position of the computer mouse (although we analyzed only the (x) projection of the mouse on the response scale). The position of the computer mouse was measured every 250 ms (1/4 s).

### Procedure

Respondents were informed that the amount the person being tested would receive for participating in the experiment was not fixed in advance and would depend on what happened in the study (“You can earn up to 5 PLN but you can also earn nothing”). The experimenter asked the participant to take a seat in front of his/her computer and to follow the instructions on the screen. Subjects in the study performed several “tasks.” They:

• learned about the use of the program interface
• answered demographic questions, about their mood and state of finances
• completed a study of social orientation (Grzelak, 1982).

The participants did not receive any feedback about their social orientation. They simply filled out the questionnaires.

After performing the above-described tasks (filling out the questionnaires), information about the end of the study and remuneration for the participant’s participation appeared on the computer screen. The participants found out that they would be paired with another person and that they had to divide the wages (5 PLN) between themselves. In fact, the “other person” did not exist. They were led to believe that the task they had performed was also completed by another person sitting in another room, located in a different part of the laboratory’s building. They were informed that there was no opportunity to meet the other person or to get to know who this person was. The participants performed seven tasks and were led to believe that “the other person had done” 10 tasks. The hypothesis stated that pro-self decisions are preferred in the first seconds of the decision making. A situation in which the amount of workload was unequal was created in order to test this hypothesis. If the workload was equal, the simple rule of equal division would be favored. If, on the other hand, the participants had a higher workload, the situation would not be diagnostic, as the pro-self decision would be the result of arithmetic. As a result (with this hypothesis stated), the alleged partner always performed 10 tasks and the participant did 7.

The next step was a draw as to which of the two divisions (the participant’s or the other person’s) would be taken into consideration. Every participant had the right to distribute the money as there was no “other person.” After the draw process the subjects saw a message on the computer screen that they were “supposed to split the money between themselves and their partners and this task will start in 10 s.” At the same time, the scale on which they distributed the money appeared below this message, i.e., at the bottom of the screen. The time period was chosen after having conducted the pilot study. The participants had no time to think about their decisions, as this was a large amount of information. The participants were supposed to become acquainted with the instructions and the scale in the first 10 s.

After 10 s (from the first appearance of the scale on the computer screen and message about the task) they were asked to point to the middle of the scale. From this time onward, spontaneous mouse movements were measured. Then they were asked to think about their decisions, to choose a final option on the scale and to click on it (these requests also appeared on the computer screen).

Clicking on this scale, the subjects decided how much money from a pool of 5 PLN they would spend on themselves and how much on the other person. In addition, the experimental group was distracted: a flashing screen and elapsed time. A total of 40 s was the limit for the answer under the distraction condition. Subjects could respond at any time before the time limit. There was no limit under the no cognitive load conditions (they could deliberate for as long as they wanted). At the end of the test the participants received the money they had “won.”

### Results

First, spontaneous mouse movements were analyzed. We used raw data from the time the participants were asked to point to the middle of the scale until they clicked on it. Variance analysis with repeated measures was then calculated.

A significant main effect was observed for the temporal dynamics of mouse movements. With the passage of time the participants decreased the amount of money that they took for themselves (“diminish selfishness”), *F*_(48,1008)_ = 2.19, *p* < 0.001, η^2^ = 0.09.

There was also a significant main effect of the availability of cognitive resources. During the decision-making process, and before clicking, people granted more money to themselves when they were in the distractor group than when they were in the control group, *F*_(1,20)_ = 5.48, *p* = 0.03, η^2^ = 0.22.

**FIGURE 2:**
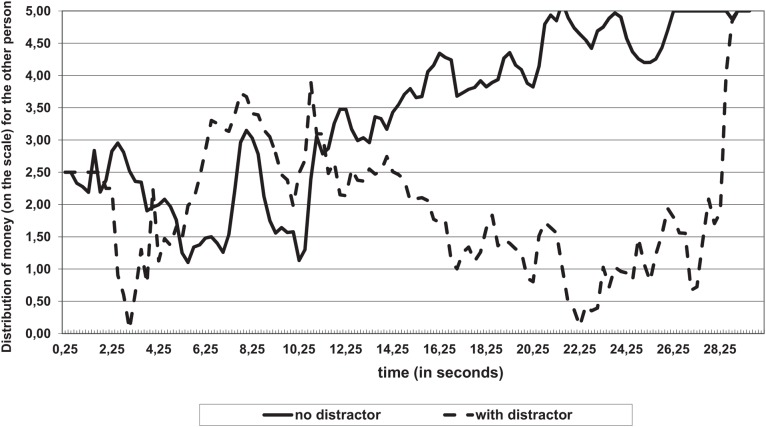
**Distribution of money (on a scale) as a function of time and the conditions of the distribution (with or without a distractor)**.

A key finding was the result of significant interaction (Distribution of money by Time ^∗^ Resource availability) between time and the availability of cognitive resources *F*_(48,960)_ = 1.46; *p* = 0.04, η^2^ = 0.07 (see **Figure 2**).

In the control group (no distractors), a slight gradual increase in altruism was found (participants distributed more money to others). In the experimental group this change was strong and had a curvilinear structure.

The differences (between groups with a distractor and without it) were stronger for participants who took more time to make a decision (from 17 to 28 s).

The subjects could respond at any time before the 40-s time limit. They differed in the time period of making spontaneous mouse movements; some of the participants made a decision more quickly, whereas others took longer. The participants made a decision more quickly in the distractor group (*M* = 18,4 s) than in the control group (*M* = 28,77 s), *F*_(1,65)_ = 15.36; *p* < 0.01, η^2^ = 0.19.

The data was normalized as a second step of analyzing the results. The aim of the normalization was to compare different parts of the spontaneous decision process. To better understand this idea, we can imagine that the whole process of making a decision takes 100% of the time, and we can divide it into 10% intervals, e.g., we can check the mouse movements of the respondents during the first 30% of the time for thinking and 20% of the time before the end. Data was normalized after the following formula:

length of time from the start of measuring to the finish (click) for every person = *T_i_*
size of a normalized time unit for person y *i* = *ti* where *t_i_* = *T_i_*/*t* of normalized time *k* = {0%; 10%; 20%; ……; 100%}
Position of the computer mouse cursor for each person = *X*
Position of the computer mouse cursor in normalized time k for each person *i* = S*_ki_*
S*_ki_ = X_i_*|*t*_i^∗^_ *k.*

**FIGURE 3:**
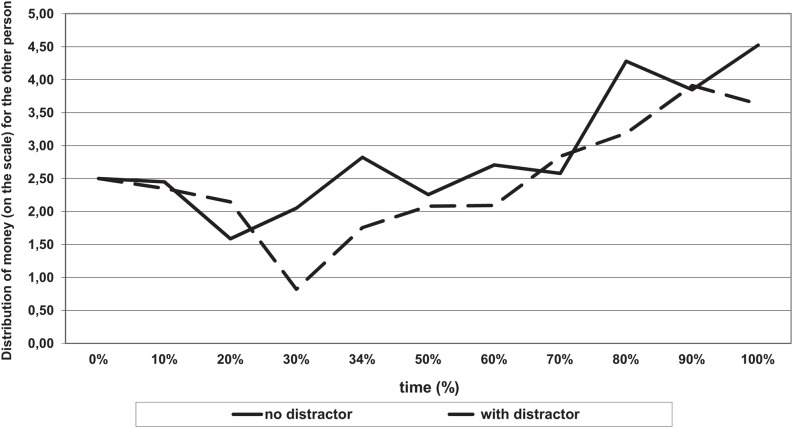
**Time-normalized responses.** Distribution of money (on a scale) as a function of time and the conditions of the distribution (with or without a distractor).

The normalized results showed a similar pattern as the raw data (see **Figure 3**). A significant main effect was observed for the temporal dynamics of mouse movements. With the passage of time the participants decreased the amount of money they took for themselves, *F*_(10,650)_ = 6.74, *p* < 0.001, η^2^ = 0.09. Spontaneous mouse movements on the response scale revealed egoistic weighing of the decision (splitting the money between the partners and themselves) in about the first 30% of time of thinking, after which it became more altruistic; for the square function *F*_(10,650)_ = 19,06, *p* < 0.01, η^2^ = 0.22. This pattern was similar under conditions of low vs. high cognitive resources availability. There was no significant interaction (Distribution of money by Time ^∗^ Resource availability) between time and the availability of cognitive resources *F*_(10,650)_ = 1.46; *p* = 0.054, η^2^ = 0.01.

### Discussion

The mouse paradigm allowed us to show the dynamic process of making a decision in the Dictator Game and gave some insight into the academic debate as to whether people are intuitively inclined to cooperate so that reflection will cause them to behave selfishly. We observed that in the first few seconds the participants may be more selfish. Then, after the first few seconds have passed, their spontaneous mouse movements became more altruistic. The selfish remuneration division was noted especially with the distractor. There was no such significant difference under conditions of low and high cognitive availability in the normalized data. The pattern of results (first egoistic choices, then altruistic ones) was similar in the two groups, but under distraction conditions the participants made a decision more quickly than in the control group. These differences in the time reactions explain the significant effects in the raw data but were reduced after normalization, which led to an extension of the pattern of reactions under the distractor conditions and a reduction of the temporal dynamics in the “high availability” group.

A low level of altruism with limited resources in a specific situation was observed, among others, in a classic experiment by Darley and Batson (1973). In this experiment it was a lack of time that modified the desire to help students at the seminary (going to a seminar devoted to “The Good Samaritan”). Only 10% of the tested seminarians who were informed by the experimenters that they were late for class in another building helped the person in need that they met on the way. Yet as many as 63% of the “not-in-a-hurry” seminarians helped the person. It can be assumed that in this study the standard related to the parable of the Good Samaritan was not automated enough to influence the behavior in the absence of resources (time). This standard was important (along with the entire system of values instilled in the seminarians) only with the action at the controlled level, i.e., in the absence of a cognitive demand. The behavior of the majority of people “in a hurry” can be interpreted either as yielding to a generally automated heuristics of hurrying up (“when in a hurry I will not help”) replacing the detailed guidelines of behavior in a given situation or (at least in some people) as yielding to automated (and not revealed at a conscious level) egoistic orientation (acquired during one’s life or innate, i.e., originally automatic).

This study does not settle assumptions about the egoistic or altruistic nature of a human being. The results confirm that the participants divided the resources and favored themselves despite the unequal workload situation. In the first seconds the fact that the participants had less of a workload did not matter, as they acted according to the activated self-interest goal. With time, information concerning the workload could have been considered while dividing the money.

Our results show that conflicting data concerning the impact of limited cognitive resources on altruistic behavior may be related to the dynamics of behavior, i.e., the most selfish choices would be in the first spontaneous reactions. Then the choices were more altruistic because the norm of fairness could have been applied, i.e., if the subjects have them and the time to use them. These results correspond with research by Rand et al. (2012). The studies measured the final decision, not the process of its being made. Therefore, this study may show that tracking the first movements gives the opportunity to observe how the automatism of meeting one’s own needs is replaced by the norm of fairness. The results also have an alternative interpretation. People are not selfish and are altruistic in nature, but their reaction depends on the situation, i.e., the salience of cues in the performed task. The Dictator Game and a chosen distraction task, i.e., information about the possible loss of money, could have activated actions focused on satisfying self-interest. Then people considered the information that the participants had performed fewer tasks then their partners and tended to give them more money. Information about partners filling out more questionnaires then could lead to the application of cooperative heuristics and to giving more money to the partners.

The participants were supposed to become acquainted with the instructions and the scale in the first 10 s. We consider this a limitation of the interpretation of the study’s results, as some participants might have processed the information beforehand. Therefore even during reading the instructions the participants were distracted (the flickering of the screen and the information about the time pressure). In Study 2 a training task was added to avoid the differences in the time length between the groups caused by their becoming acquainted with the procedure.

## Study 2

Recent polls that were conducted in Poland show that the proportion of people declaring prejudice against Jews (the ethnic minority, not the religious group) is decreasing (Maliszewski, 2011). This could be partially the result of the more widespread norm of political correctness and internalization of egalitarian values. Hence, the declared attitude may be the result of inference about Jews on the basis of the respondents’ values and norms. When the cognitive resources of an individual are limited, it is difficult to take into account the overall situation and decide what is and what is not incorrectly assessed. In such situations the most automatic processes are selected. Their source may exist in the memory patterns of associations, i.e., introspectively unidentified (or inaccurately identified) traces of a past experience, thus implicit attitudes. Studies in which attitudes were measured by the IAT confirmed that Polish students preferred Poles as compared to Jews on an implicit level (Maliszewski, 2011) despite the fact that they declared no difference in attitudes between them. It was important to study the conditions under which people may apply to behavior those implicit attitudes.

Kraus’ meta-analysis Kraus’ (1995) of 88 studies on explicit attitudes and behavior showed a moderate correlation of *r* = 0.38 between attitude and behavior. Greenwald and Banaji (1995) expected that implicit attitudes would predict these behaviors which explicit attitudes could not. The results of the first studies, however, did not confirm these expectations. It turned out that the implicit attitudes, measured by the IAT, were less related to behavior than explicit attitudes (Karpinski and Hilton, 2001). The reason for obtaining the results of a low attitude and behavior relationship was that the behavior measured was not specific for this particular type of attitude (cf. Dovidio et al., 1997; Marsh et al., 2001; Perugini, 2005). McConnell and Leibold (2001) demonstrated that implicit attitudes are strongly associated with “spontaneous” behavior, which is undertaken without effort and control of the entity, e.g., the length of the conversation or eye contact. The explicit attitudes were highly correlated with “thoughtful” behaviors, which are the result of considering an existing situation.

Studies showing a stronger relationship between implicit attitudes and spontaneous behaviors have suggested that the goal of the research should not be to determine whether the implicit attitude is a good predictor of behavior but rather under which specific conditions it influences behavior. According to Wilson et al. (2000), implicit attitudes are active when a person does not have the resources or motivation to control its impact. On the other hand, when he or she has the cognitive resources and motivation to bring the attitude from memory, that person’s behavior is based rather on the explicit attitude than the implicit one. These assumptions were tested in Study 2. In this experiment the participants evaluated an image that had allegedly been painted by either a Pole or a Jew. The implicit attitude could manifest itself when one was thinking about an evaluation of the image and when one moved the mouse but did not know that his/her reactions were being recorded. It was expected, therefore, that in a situation of low cognitive resource constraints the individual would strongly favor Poles in comparison to Jews. It was assumed that spontaneous evaluations would be more favorable to their own group and less favorable to the foreign group (i.e., in the case of Jews).

### Method

#### Subjects

A total of 79 participants took part in the study. These were students of Warsaw University (excluding the Faculty of Psychology) living on the university campus (age *M* = 19.73; *SD* = 0.828).

#### The Study Design

Independent variables: (1) conditions of attitude activation – cognitive resources availability (without distraction) vs. limited cognitive resources (when performed tasks are accompanied by a distractor); (2) explicit attitude measured before the experimental manipulation; (3) implicit attitude measured before the experimental manipulation; (4) ethnicity of the painting’s author – a Pole or a Jew. The dependent variable was a spontaneous (figuring out) and final (click) evaluation of the painting.

#### Tools and Materials

An indicator of an explicit attitude toward Poles and Jews was the temperature that the person indicated on the feelings thermometer (from -50 to +50°C; the higher the temperature, the more positive the attitude; details, please see Greenwald et al., 1998).

**FIGURE 4:**
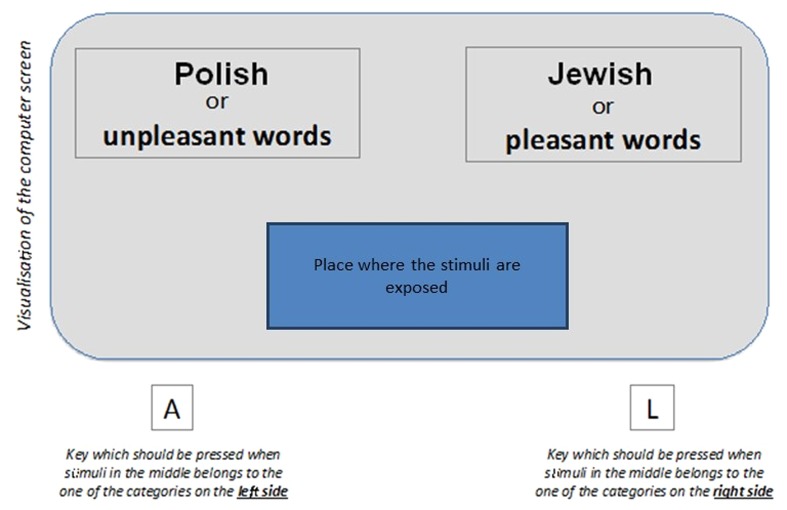
**Example of the Implicit Association Test (IAT) to measure implicit attitudes toward Poles and Jews**.

Implicit attitudes were measured using the IAT (the applied procedure by Greenwald et al., 2009). It was conducted individually with the use of a computer. The person examined a series of stimuli and classified them into appropriate categories using two keys assigned to the response (“a” and “l”). Name categories were displayed in the upper right- and left-hand corner of the monitor, and the stimuli appeared on the screen (please see **Figure 4**).

The computer recorded the time and accuracy of the response. The test consists of seven blocks: three out of the seven tasks in the IAT are *single categorization* tasks which are practice blocks where the participants are exposed to stimuli and learn to respond appropriately. The other four blocks are *combined tasks*, where four categories are categorized simultaneously (e.g., Polish, “pleasant” and Jewish, “unpleasant” words; see **Table 1**).

**Table 1:** Schematic illustration of the Implicit Association Test (IAT).

Task	1	2	3 and 4	5	6 and 7
Task description	Category of affective words	Category of words which were the subject of the study	Complex category – words from the 1st and 2nd task	Category of words which were the subject of the study with an inversed order	Complex category – words from the 1st and 2nd task with an inversed order
Category name	<Unpleasant	<Jewish	<Unpleasant	Jewish>	<Unpleasant
			Jewish		Polish
	Pleasant >	Polish>	Pleasant	<Polish	Pleasant
			Polish>		Jewish >
Stimuli words – objects which are classified in a particular category	sun>	Polish national	Polish	<Catholic cemetery	<Catholic cemetery
	<death	anthem>	emblem>	a child in a	Israeli national anthem (Hatikvah) >
	holidays>	<Israeli national anthem (Hatikvah)	<Israeli flag	skullcap>	<illness


	<illness		sun>		
	<poison		<death		
	joy>				


*The arrow next to the category represents the side of the computer screen next to which it was displayed. The arrow next to the stimuli word (examples) represents the category which the world should be classified into. Columns with the gray background are crucial for the study.*

The first two tasks are simple categorizations in which a person assigns stimuli to one of two categories:

(a) In the first task – pleasant (sun, holidays, joy, love, pleasure) vs. unpleasant words (illness, death, poison, evil, failure);
(b) In the second task – pictures symbolizing target categories; Jewish (five stimuli, e.g., the flag and emblem of Israel) vs. Polish (five stimuli, e.g., the flag and emblem of Poland).

Blocks 3/4 and 6/7 constitute complex categorizations and are a combination of the first two tasks. They consist in assigning on-screen stimuli into one of four categories, arranged in pairs in the upper corners of the screen (combined categories of tasks 1 and 2, e.g., Unpleasant or Jewish vs. Pleasant or Polish). They are also divided into practice blocks (3 and 6; 30 categorizations of stimuli) and crucial blocks (4 and 7; 40 categorizations of stimuli). After the third task (e.g., Unpleasant or Polish vs. Pleasant or Jewish), the sides of the test category are reversed (e.g., Unpleasant or Jewish vs. Pleasant or Polish). The order of these blocks was counter-balanced across the participants. The analysis uses the reaction times from the combined tasks. The difference in the average response time for the initial combined blocks (3/4) and the reversed combined blocks (6/7) is an indicator of implicit attitudes.

In interpreting the IAT it is assumed that people are able to give the same response to items in two categories more quickly and more easily when those categories have the same evaluative value (e.g., both are negative for prejudiced respondents, unpleasant and Jewish) than when they are not (e.g., one is positive – Polish and one is negative – unpleasant).

**FIGURE 5:**
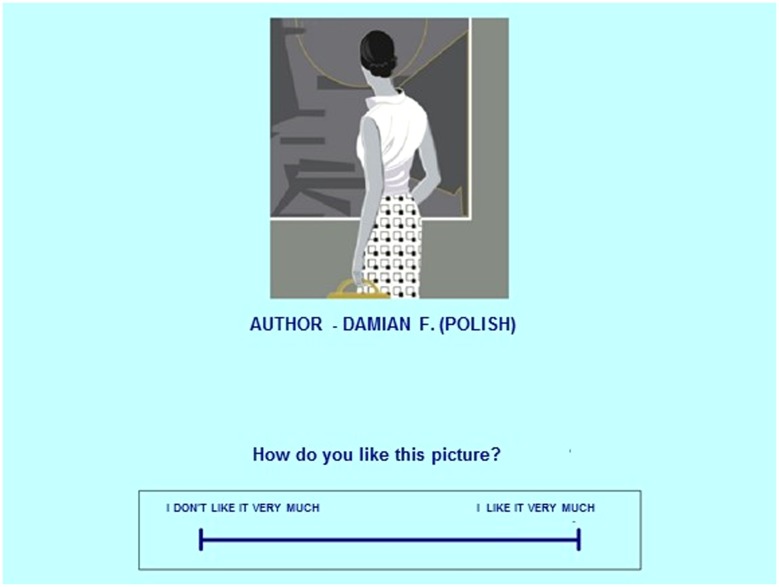
**Screen used to assess the image by a Pole/Jew**.

The second tool that was used in the study was the “mouse method.” Respondents evaluated an image that was placed in the center of the screen. Beneath it was a signature – the name and nationality of the author (e.g., Damian F., Polish, Anna T., Polish, David B., Jewish, Martha S., a Jewish woman). The gender of the image’s author was the same as the person being examined (e.g., David for a male, Anna for a female). At the bottom there was a continuous scale marked “very much dislike” (on the left), “like very much” (right) (see **Figure 5**). As in the first study, the results were the positions of the cursor on the X axis measured from the middle of the scale.

### Procedure

The subjects were recruited from a student dormitory. First, they filled out a questionnaire which included questions about their age, gender and nationality, as well as their explicit attitude toward both Jews and Poles. After completing the questionnaire, the participants were seated in front of a computer. Then the implicit attitude was measured using the IAT. After that, behavior was studied by the “mouse method.” The subject was told to imagine a conference which would bring together Jews, Germans and Belarusians living in Poland as well as Poles. The aim would be to evaluate three works that had been awarded at the conference. First, a painting by a German artist would be evaluated in a practice trial. Then the true purpose of the study would take place – a student evaluated two images whose authors were a Pole and a Jew (the order and image associated with a Pole and a Jew were counter-balanced across participants). Spontaneous mouse movements were measured from the appearance of a new painting (there was no instruction to point the computer mouse to the middle of the scale; the cursor remained in the place where the subjects had clicked in the previous task). Depending on the group, the task was performed under the following conditions: distractor absorbing the cognitive resources (the participant was asked to count backward from 100: 100, 99, 98, etc.), or with no distractor (control condition). There was no time limit for this task. The participants could see the (x, y) position of the computer mouse [but only the (x) projection of the mouse on the response scale was analyzed]. The position of the computer mouse was measured every 500 ms (1/2 s). After the study, the person received 5 PLN in remuneration.

### Results

**FIGURE 6:**
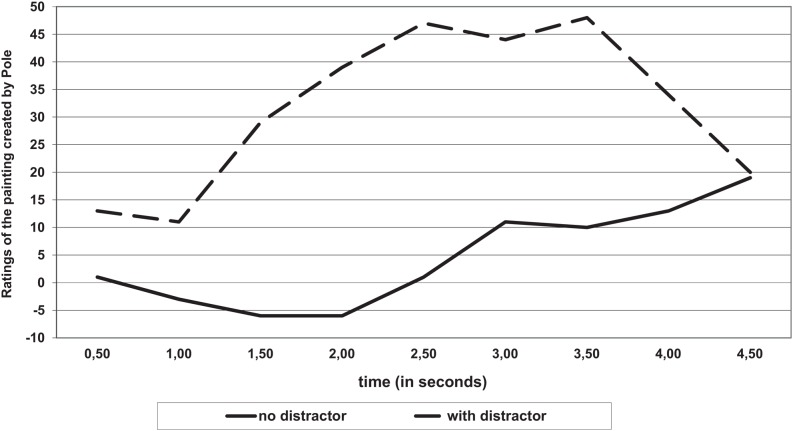
**Ratings of a painting depicted by a Pole (graphic scale -90 mm, +90 mm , where 0 is the center of the scale) as a function of time and the evaluation (with or without a distractor)**.

First, spontaneous mouse movements made while thinking about an evaluation of the paintings by a Pole were analyzed (see **Figure 6**). Then variance analysis was conducted.

Ratings of the painting depended on the conditions of cognitive resources availability, *F*_(1,38)_ = 4.44, *p* = 0.04. η^2^ = 0.10.

There were no significant changes when the dynamics of the evaluations in time were taken into consideration, both with and without the distractor (*p* > 0.05). The dynamics of the evaluations with the distractor, however, were close to significant *F*_(1,14)_ = 3.85, *p* = 0.07; η^2^ = 0.19. Therefore, *post hoc* pairwise comparison tests were used (LSD^∗^ method of the smallest significant difference) to compare the evaluation of the Pole’s image with and without the distractor. Groups with 1.5–3.5 s were significantly different (*p* < 0.5).

For example, in the first moments the person in the distractor group favored the image by a Pole (up to 3.5 s), but with time the assessment became less favorable.

Next, multiple regression analysis was used (a step backward selection algorithm) to find the best predictor for movements of the mouse. The initial model consisted of: explicit attitudes toward Poles, implicit attitudes, task conditions (distractor vs. no distractor), and the interaction of variables (conditions ^∗^ explicit attitudes, conditions ^∗^ implicit attitudes). Such an analysis was performed for each time point (measured every 0.5 s) and for the final evaluation of the painting.

The only significant (*p* < 0.05) predictor of spontaneous behavior (mouse moves while pondering) was the interaction of implicit attitudes and the conditions for the task in 2 s (η^2^ = 0.08); 2.5 s (η^2^ = 0.14); 3 s (η^2^ = 0.07). In the remaining time and the final assessment (click) there were no significant effects of the independent variables. Under the condition of low cognitive resources availability (with a distractor), the stronger the positive implicit attitudes the participants had toward Poles (in-group favoritism), the more preferably the image of the Pole was assessed, e.g., 2.5 s, η^2^ = 0.27. The relationship was reversed under conditions without a distractor, i.e., the more the participants favored the in-group (Poles), the less favorably they evaluated the image of the Pole, although this relationship was significant only at the level of the trend, 2.5 s, η^2^ = 0.07.

**FIGURE 7:**
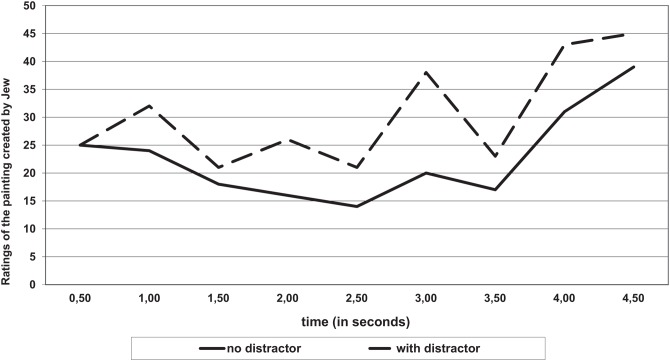
**Rating of an image by a Jew (graphic scale -90 mm, +90 mm, where 0 is the center of the scale) as a function of time and the evaluation (with or without a distractor)**.

An interesting behavior dynamic was also observed when the participants evaluated the image painted by the Jew (both among the participants who were and were not distracted). Mouse movements during the first 2 s expressed the tendency to give a worse evaluation of the Jew’s image (decreased ratings, **Figure 7**); however, with time the image evaluations were more favorable, *F*_(1,41)_ = 7.5, *p* < 0.01, η^2^ = 0.15.

The factors that affect the mouse movements during the assessment of the image painted by a Jewish person and the final assessment were examined with regression analysis. The model contained elements that were analogous to the previous analysis, i.e., an explicit attitude toward Jews, implicit attitude, the conditions of the task (distractor vs. no distractor), as well as the interaction of variables (conditions ^∗^ overt attitude, conditions ^∗^ implicit attitude). The only significant predictor of behavior (*p* < 0.05) was the interaction of explicit attitude and task conditions: 2 s (η^2^ = 0.07); 2.5 s (η^2^ = 0.06); 3 s (η^2^ = 0.10); 3.5 s (η^2^ = 0.16).

In the remaining time and the final assessment (click) there was no significant effect of the independent variables highlighted. An additional analysis showed that under the conditions without the distractor the evaluations were consistent with the explicit attitudes: the more positive the attitude participants had, the more they liked the Jewish image (2–4 s), e.g., 3.5 s, η^2^ = 0.12. In contrast, with the distractor the relationship was reversed: the more the person had a positive explicit attitude, the less he/she preferably assessed the image by the Jewish person (from 2 to 4 s), e.g., 3.5 s, η^2^ = 0.19.

### Discussion

The pattern of results turned out to be more complex than expected. The hypotheses were supported primarily under conditions with the distractor and when participants were evaluating the image painted by a Pole. It was observed that between the 2nd and 3rd second the Polish artist was favored (the respondents moved the mouse to the right). Additionally, we studied to what extent those spontaneous assessments (mouse movements before the decision) were associated with implicit attitudes (IAT). The results confirmed that the IAT predicted spontaneous mouse movements during the evaluation of the image painted by a Pole.

When there was no distraction and the Pole’s painting was judged, paradoxically, it was found that the stronger the implicit in-group favoritism the participants had, the worse they assessed the Pole’s image. Fazio et al. (1995) received analogous results in the relationship between attitudes of white Americans against African-Americans (as measured by affective priming) and declarations. It turned out that among those white Americans who had egalitarian values, the stronger the implicit prejudice they had against African-Americans, the more tolerant the attitude toward them they declared. It can be assumed that students in Experiment 2 were guided by the principle of egalitarianism. In order to inhibit an undesirable, i.e., for them, implicit in-group preference, the participants assessed the Pole’s painting worse probably on the basis of compensation.

There was no relationship between the IAT and a spontaneous evaluation of the image painted by a Jew. The lack of this dependence may be associated with the properties of the IAT method. It has two categories (Polish vs. Jewish) and it is impossible to evaluate how much of an effect the IAT is an expression of in-group preference (Polish) and how much of out-group discrimination. In this study the IAT effect could be primarily an expression of implicit in-group favoritism (Polish) and, therefore, significant correlations were obtained only with the assessment of the Pole’s image. In order to verify this hypothesis, it would be helpful to use one category in the IAT, or the GNAT method (Nosek and Banaji, 2001).

Noticing the impact of implicit in-group favoritism (Poles) and no significant out-group derogation also has an alternative explanation. Favoring the “in-group” is an automatic behavior that would ensure the survival of the individual because of it one can always count on support from members of one’s own group. Another consequence of in-group favoritism is to discriminate against the out-group, which is not always present (Perdue et al., 1990; Otten and Moskowitz, 2000). Discrimination toward the “out-group” can be more thoughtful behavior. Therefore, only explicit attitudes influenced this behavior.

If this interpretation were correct, it would mean a significant limitation associated with the use of the “mouse method.” Studies have shown that implicit attitudes toward Jews may manifest themselves in an automatic way, including in non-verbal behavior (Maliszewski, 2011). The “mouse procedure” would reveal, however, only the strongest automatic processes associated with in-group favoritism.

The reason for the smaller significant effects than expected may also be related to the confounding variables of the same method. In Study 2 the mouse cursor was not placed in the middle of the screen. It was at the point where the subjects had finished the previous task. This problem will be solved in subsequent studies by appropriately placing the board prior to the task. Then the subject will be asked to move the cursor to a designated point and will click on it (this point will be located in the middle of the scale of the task to the right).

In Study 2, normalization of spontaneous mouse movements gave a similar pattern of results as the raw data, so there was no need to describe it. The decisions were much quicker than in Study 1, because in Study 2 the participants had a trial task to complete and the task was simpler – the evaluation of the painting (the amount of information to process) than the division of the money after the task.

## General Discussion And Summary

The experiments conducted here confirm that the first spontaneous movements of a computer mouse may reveal selfish behavior and might be an expression of in-group favoritism.

Study 1 showed that in the first seconds the participants split the money in a selfish way, but with time they behaved more altruistically. These results can be interpreted in terms of complex systems (cf. Vallacher and Nowak, 1994). When subjects adapt to the situation of the distribution of money (in the first few seconds), the role of the simpler mechanisms is crucial, e.g., favoring egoistic solutions. In this scenario normative and moral structures are probably not available and the subjects behave in a selfish manner. With time, however, the importance of more complex mechanisms, such as norms and values, could increase. Consequently, in the following seconds the subjects behave in a more altruistic manner. This process is slower when the distractor works. Most probably the distractor absorbs the cognitive resources of the participants and they cannot apply them to distribution of wealth based on the standards and values of people. Similar results can be observed in classic studies by Darley and Latane (1968) on the determinants of support behavior, i.e., where external factors such as, for instance, haste, or the number of people or uncertainty, proved to negatively modify the desire to help. This may prove the impact of automated, coarse heuristics counteracting helping in such situations in contrast to the situation without haste, with a small number of other people, tending to consciously aid activities. Garcia et al. (2002) showed that priming a group context by asking participants to imagine being in a group reduced the willingness to help in a subsequent task (i.e., minutes volunteered for another experiment) in line with an (implicit) bystander effect.

The prevailing amount of research supports the hypothesis in seeming opposition to Study 1, i.e., that humans are altruistic when responses are required with time pressure or/and low cognitive resources (Rand et al., 2012; Lotz, 2015). We claim that it is only a superficial contradiction and that there is no conflict between automatic and deliberative responses in the context of cooperation. Study 1’s results show that people on an automatic level can be selfish or altruistic, and that this depends on the time of reaction. At first the subjects behave selfishly because the primary purpose of pursuing the self-interest is operating (van den Bos et al., 2006). Later, norms of fairness may be applied (if the task solicits them and they are available).

People are not selfish or altruistic by nature. Their reaction depends on the situation: the salience of cues in the performed task but also on the availability of their heuristics pro-selfs vs. pro-socials. Perugini et al. (2011) assumed that, “the perception of certain features of the task (the environment) can solicit a heuristic (or behavioral schema) as long as there is an association between the environmental cues and some related motivationally-imbued cognitive activities,” e.g., dark rooms promote egoistic behaviors (Zhong et al., 2010), clean scents lead to altruistic behavior (Liljenquist et al., 2010), and bright cues are perceived intuitively as well (Meier et al., 2004).

The theoretical framework which assumes a similar approach including both the context and individual differences is the Social Heuristics Hypothesis (SHH, Rand et al., 2014; Rand, 2016). Its basic assumption is that “behavior that maximizes payoffs in the long run is automated as a social heuristic,” i.e., persons who are able to automate both altruistic and selfish behaviors. However, Rand (2016) assumes that generally “intuition should favor cooperation, given the pervasiveness of mechanisms (e.g., repeated interactions, concerns about reputation) that make cooperation advantageous in daily life in the long run.” On the reflective level, people take into consideration different features which can inhibit the application of the intuitive response, which is usually cooperative. Rand (2016) argues that “deliberation will favor either cooperation or non-cooperation depending on the individual’s explicit beliefs about which behavior will maximize his or her payoff.”

### Dynamics of Implicit Attitudes’ Influence on Behavior

A meta-analysis conducted by Greenwald et al. (2009) of 61 studies on the relation of attitude to behavior revealed that the implicit attitude is a better predictor than the explicit attitude of behaviors that are socially “sensitive” and those that are difficult to control, e.g., in the areas of stereotypes and prejudices (*r* = 0.25 and *r* = 0.13). In contrast, the explicit attitude has more influence than the implicit attitude on consumer behaviors (*r* = 0.71 and *r* = 0.40) and election (*r* = 0.67 and *r* = 0.41).

The phenomenon of in-group favoritism is incompatible with social egalitarian values. Many studies on the minimal group paradigm showed that a random division of people into two groups, e.g., possessing a red or green pen, was enough to create an in-group favoritism reaction (Tajfel, 1978). Ashburn-Nardo et al. (2001) showed that even a random assignment of respondents to groups labeled as preferring one of two fake painters (Quan or Xanthie) was enough to observe implicit preference for one or the other painter in the IAT task, e.g., those who were labeled “preferring Quan” also preferred Quan more than Xanthie at an implicit level. Because the “preference label” was assigned randomly, it had nothing to do with the actual preference for a particular painter but had a much more basic character.

Study 2 also showed in-group favoritism measured by the IAT. Polish students preferred Poles to Jews at an implicit level. At the explicit level their attitudes were not significantly different. The implicit in-group favoritism predicted spontaneous reactions during an evaluation of a painting made by a Pole. This relationship between implicit attitudes and behavior was dependent on the participant’s availability of cognitive resources, type of reactions (spontaneous vs. deliberative) and its time (first reaction or after a few seconds).

Activation of the implicit attitude is inevitable, but its application to generate behaviors depends on a person’s cognitive resources (Devine, 1989; Gilbert and Hixon, 1991; Blair and Banaji, 1996). When resources are not available, and the person cannot think over his or her behavior, then the implicit attitude is activated. In a study by Friese et al. (2008), cognitive resources were experimentally manipulated. It turned out that in the group with limited cognitive resources the amount of chips eaten was associated with implicit attitudes. However, in those individuals whose capacity was not limited, a significant relationship with explicit attitudes was observed. Similarly, in the following study, when the evaluation distractor was activated, assessment was related to implicit attitudes.

It should be noted, however, that even with limited cognitive resources, the impact of implicit attitude concerned only spontaneous behaviors (the first moves of the mouse), and not a final decision or final assessment (a click on the scale). Implicit attitudes are associated with “spontaneous” behavior that is difficult to control (cf. Dovidio et al., 1997; Marsh et al., 2001; Perugini, 2005).

Another element is the reaction time. As the mouse movements of the respondents showed, the first reactions were associated with implicit attitudes, then the explicit attitude would take over the regulatory role whose application requires more time. Studies using neuroimaging suggest that, depending on the moment in which certain reactions are examined, different manifestations of attitudes appear. In one of the studies (Cunningham et al., 2004), the images of African-Americans and white Americans were presented to participants for a very short period of time of 30 ms, which prevented reflection (automatic process) and for a longer period of time, i.e., 525 ms (reflective process). When the images were presented for a short period of time, activation of the amygdala (negative affect) was significantly correlated with the implicit attitude toward African-Americans (*r* = 0.79), while there was no relationship with the explicit attitude. The arousal of the amygdala is observed, among others, during an automatic response to a negative affective (Cunningham et al., 2003) and in fear conditioning (LeDoux, 1996). When the presentation time was longer (525 ms) there were no differences in the degree of activation of the amygdala but in areas of the prefrontal cortex, which indicated that there was a reflective process.

The relationship between implicit attitude and behavior may be more complex, which is shown by the impact of the implicit attitude in conditions without a distractor. Then the implicit attitude, i.e., in-group favoritism, may be inconsistent with the egalitarian values and the person can offset its effect by denying out-group preference (Maliszewski, 2011). These dynamic dependences are identified if the measurement concerns the final decision, not the process of its making. The mouse method may be helpful in its determination.

## Ethics Statement

The procedure was typical for IAT; for both test we applied (copied) procedure used by Project Implicit; more details, see https://implicit.harvard.edu/implicit/. This study was carried out in accordance with the recommendations of Ethics comittee of University of Warsaw with written informed consent from all subjects (clicking a button indicated consent). All subjects gave written informed consent in accordance with the Declaration of Helsinki. The protocol was approved by the Ethics comittee of University of Warsaw.

## Author Contributions

Conception or design of the work: NM, ŁW, HS; Data collection: NM, ŁW, HS; Data analysis and interpretation: ŁW, NM, HS; Drafting the article: NM, ŁW, HS; Critical revision of the article: NM, ŁW, HS; Final approval of the version to be published: NM, ŁW, HS.

## Conflict of Interest Statement

The authors declare that the research was conducted in the absence of any commercial or financial relationships that could be construed as a potential conflict of interest.
